# Supplementary material for: In Vivo Optogenetic Modulation with Simultaneous Neural Detection Using Microelectrode Array Integrated with Optical Fiber
Source: Sensors (Basel). 2020 Aug 13;20(16):4526. doi: 10.3390/s20164526 (PMC7472634; doi:10.3390/s20164526)
Supplement: Supplementary file 1 [file sensors-20-04526-s001.pdf]

sensors

MDPI

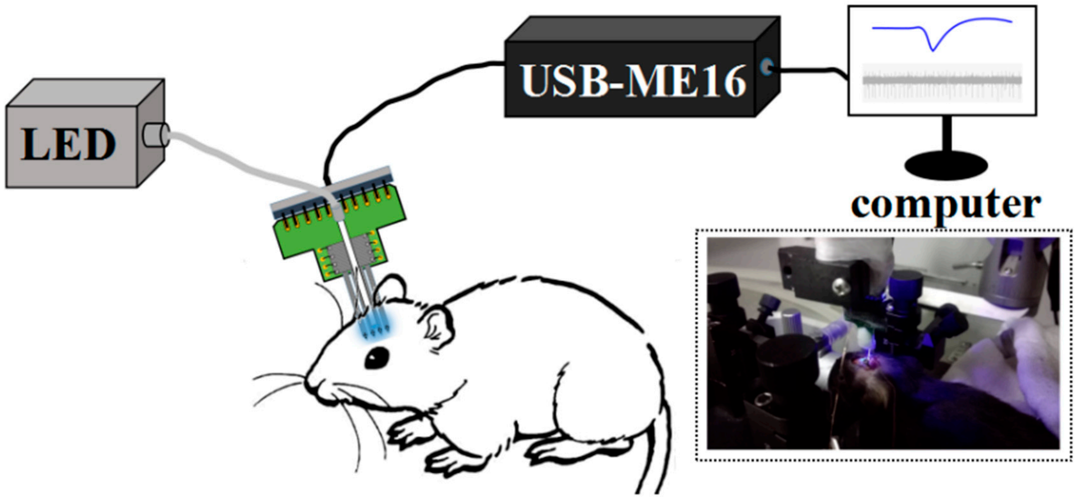

Figure 1. Schematic diagram of the experiment. The insets in the dotted boxes are photos of optical stimulation and simultaneous electrophysiology detection experiments.

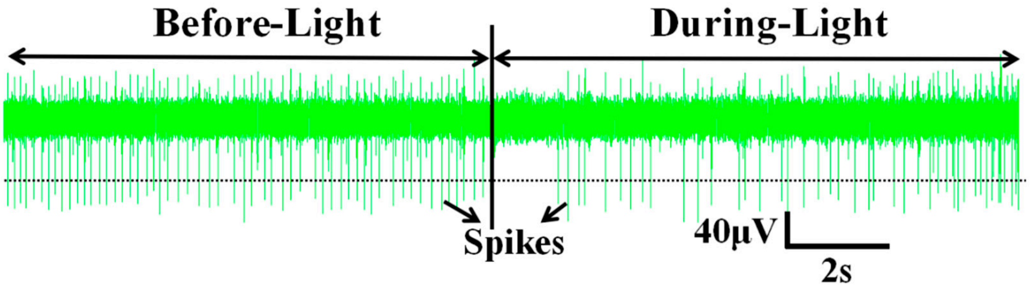

(a)

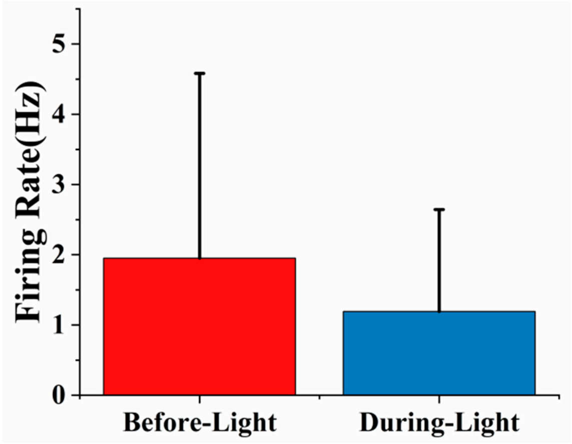

(b)

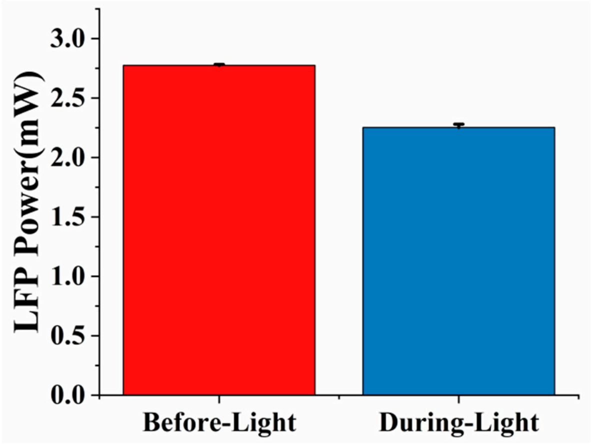

(c)

Figure S2. (**a**) The real-time recordings of electrophysiological signal before and during light illumination at a depth of 800 μm (non-viral transfection area); (**b**) the average spike firing rate of neurons before and during optical stimulation; (**c**) the average LFP power (0–30 Hz) of neurons before and during light. Error bars indicate standard deviation of 3 channels.

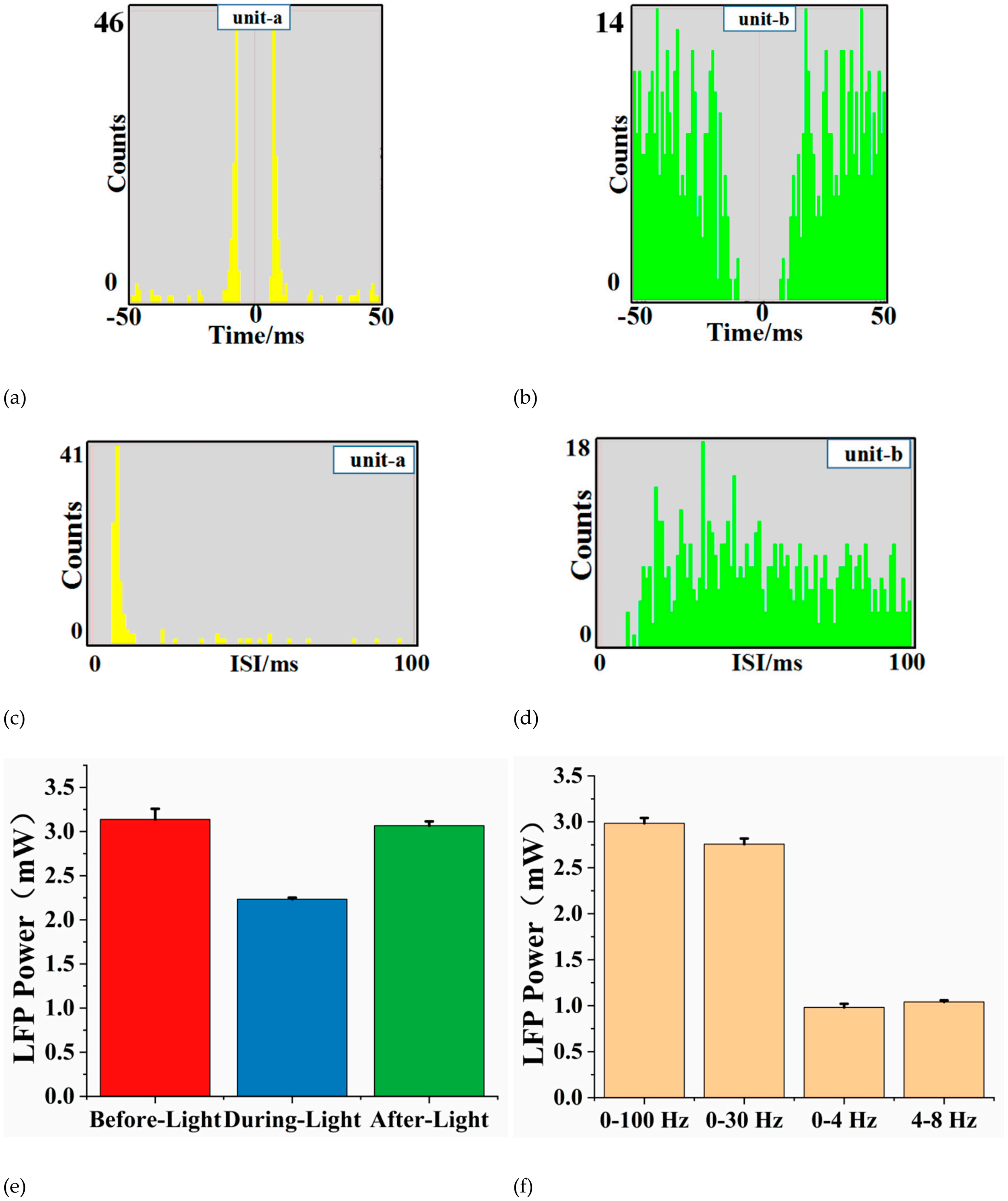

Figure S3. (**a**) Auto-correlograms of unit-a; (**b**) auto-correlograms of unit-b; (**c**) ISI histograms of unit-a; (**d**) ISI histograms of unit-b; (**e**) the average LFP power (0–30 Hz) before, during and after optical stimulation; (**f**) the average LFP power of different frequency band. Error bars indicate standard deviation of 3 channels

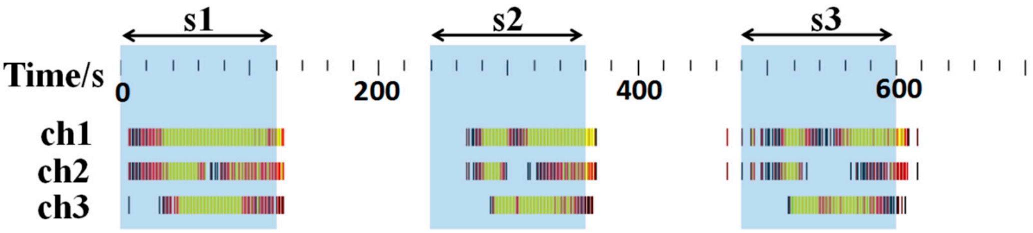

(a)

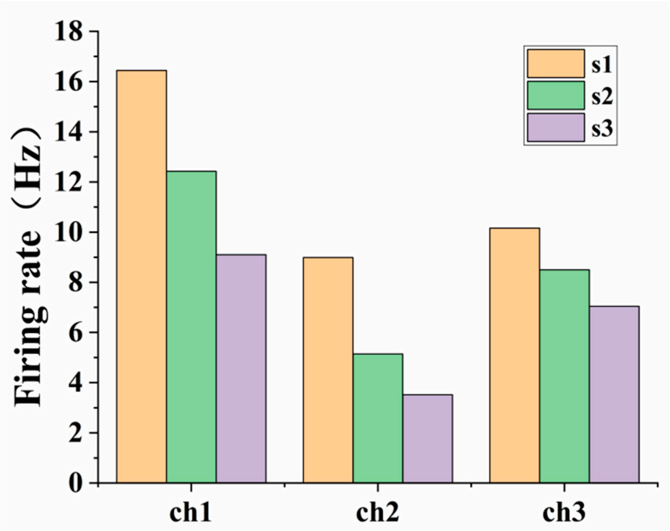

(b)

Figure S4. (**a**) The real-time recordings of spikes of three recording channels under different light stimulation patterns (s1:10 Hz, duty ratio=50%, 2 min; s2:10 Hz, duty ratio=25%, 2 min; s3:16.6 Hz, duty ratio=25%, 2 min. The shaded area is the period of light stimulation. ch = channel); (**b**) the spike firing rate of neurons detected by 3 channels under different light stimulation modes.

For simulation light intensity I (mW/mm^2) at a vertical distance *d* (mm) from the fiber tip in brain tissue, I can be estimated as (in our model, there is no coupling loss because the tip output power is known):

$$I = P_t \times \eta_{(scatter)} \times \varphi(r,d) \quad (1)$$

where $\eta_{(scatter)}$ is scattering attenuation and $\varphi(r,d)$, the geometric dispersion, is $1/mm^2$; according to the $1/d$ scattering model by Aravanis, $\eta_{(scatter)}$ can be:

$$\eta_{(scatter)} = 1 / [s(\lambda)\cdot d + 1] \quad (2)$$

where $s(\lambda)$ is the scattering coefficient for wavelength; here, $s(450) \approx s(470) = 7.2$.

the geometric attenuation could be approximated by:

$$\varphi(r,d) = 1 / \pi[r_0 + d\cdot\tan(\sin^{-1}(NA/n))]^2 \quad (3)$$

where r_0 is the radius of optical fiber (100 μm), NA is the numerical aperture of optical fiber (0.39), and n is the refractive index of brain (1.36). Therefore, we can get the final expression of estimated I as a function of distance from the fifiber tip *d*:

$$I(d) = P_t/[\pi[s(450)\cdot d + 1][r_0 + d\cdot \tan(\sin^{-1}(NA/n))]^2] \quad (4)$$

We used this expression to simulate the power density distribution with a vertical distance of 0-400 μm from the optrode tip.

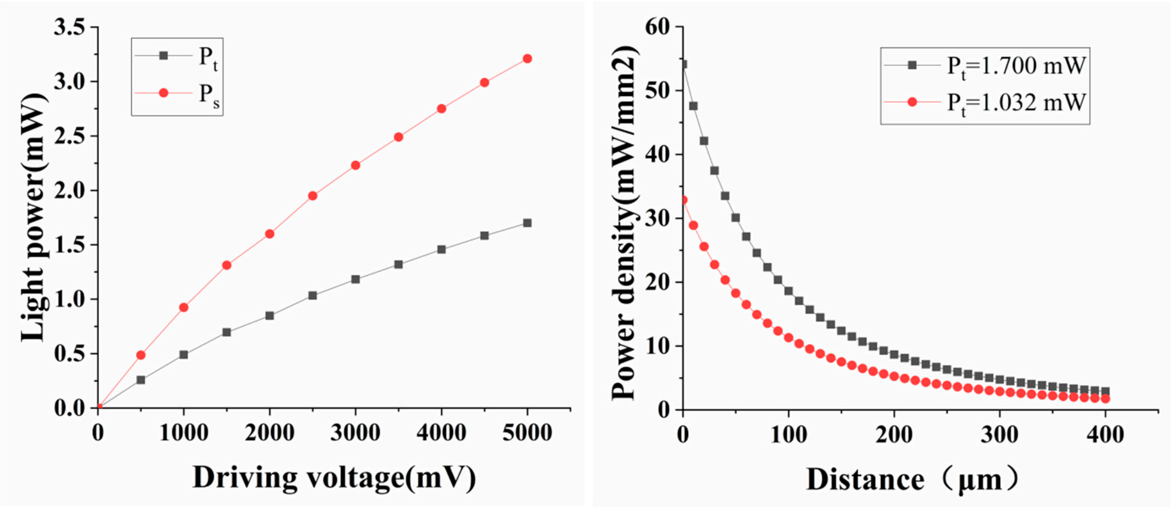

Figure S5. (a) The output power of LED light source (P_s) and the tip of optrode (P_t) under different driving voltages; (b) the simulation power density distribution with a vertical distance of 0-400 μm from the optrode tip.
